# Clinical validation of respiratory outcomes for a patch-based polysomnography system

**DOI:** 10.1183/23120541.00857-2025

**Published:** 2026-06-08

**Authors:** Holger Woehrle, Christian Viniol, Wolfgang Galetke, Georg Nilius, Christoph Schöbel, Winfried Randerath, James Leiter, Sebastian Canisius, Hartmut Schneider

**Affiliations:** 1 Lungenzentrum Ulm, Ulm, Germany; 2 Universitätsklinikum Gießen und Marburg GmbH, Department of Pneumology, Marburg, Germany; 3 VAMED Klinik Hagen-Ambrock, Hagen, Germany; 4 Klinikum Dortmund gGmbH, University Witten-Herdecke, Dortmund, Germany; 5 Universitätsmedizin Essen Ruhrlandklinik, Essen, Germany; 6 Krankenhaus Bethanien, Solingen, Germany; 7 Department of Molecular and Systems Biology, Geisel School of Medicine, Hanover, NH, USA; 8 ACliRA Consulting ApS, Tønder, Denmark; 9 American Sleep Clinic, Frankfurt, Germany; 10 Onera Health, Eindhoven, Netherlands; 11 Johns Hopkins University, Baltimore, MD, USA

## Abstract

**Study objectives:**

The Onera Sleep Test System (STS), the first wireless, patch-based, type II polysomnography (PSG) device designed to enable unattended sleep studies at the patient's home, has been introduced. This multicentre study validated data collected from the patch-based PSG against traditional PSG for the classification of sleep-related respiratory events.

**Materials and methods:**

Seven clinical sites recruited 206 participants to complete a simultaneous sleep study in the sleep laboratory with a traditional PSG and a patch-based PSG system. Three independent scorers, blinded to patient and device type, evaluated the traditional PSG and patch-based PSG recordings in accordance with American Academy of Sleep Medicine (AASM) 2020 (Version 2.6) guidelines.

**Results:**

The subjects were middle aged and mildly obese (50.9±12.3 years of age, body mass index 30.2±6.2). There was strong positive correlation between the apnoea–hypopnoea index ((AHI) using 3% desaturation or arousal rule) (0.90), apnoea index (0.89), central apnoea index (0.78), obstructive apnoea index (0.86), hypopnoea index (0.68) and arousal index (0.72) measured by the two devices. Differences in oxygen saturation (*S*_pO_2__) measurement were seen as a function of measurement site with the forehead measurements showing higher *S*_pO_2__ values. The patch-based device showed diagnostic utility (AUC ≥0.8) for AHI severity thresholds at 5, 15 and 30.

**Conclusions:**

The Onera STS patch-based PSG system demonstrated reasonably good agreement with traditional PSG systems in detecting sleep-disordered breathing events and in diagnosing obstructive sleep apnoea by the AHI.

## Shareable abstract

**A patch-based PSG device accurately identifies respiratory events and distinguishes AHI severity when validated against simultaneous in-laboratory PSG, and is a viable option for unattended home use** https://bit.ly/3KAqtEB

## Introduction

The gold standard for obstructive sleep apnoea (OSA) diagnosis is overnight attended in-laboratory polysomnography (PSG) [1]. Key signals include nasal or oral airflow, thoracic and abdominal respiratory effort and blood oxygen saturation (*S*_pO_2__), which are required to identify and distinguish different episodes of breathing reduction (hypopnoea) or cessation (apnoea). Brain activity (electroencephalography (EEG)), eye movements (electrooculography (EOG)) and muscle tone (electromyography (EMG)) are monitored to determine sleep stages and detect arousals triggered by respiratory disturbances. Other related phenomena, such as snoring patterns and changes in heart rate, are also evaluated. By quantifying the frequency, duration and severity of these events and relating them to sleep stage and body position, clinicians can determine the apnoea–hypopnoea index (AHI) thus diagnosing OSA to treat the condition effectively.

The patch-based, type II PSG device was designed to enable patients to perform self-applied, at-home sleep studies. Other patch-based innovations have been developed but do not include a full PSG signal set [2, 3]. To simplify device application, the patch-based PSG has signal acquisition differences of EEG, EMG, *S*_pO_2__, and effort and flow signals. A previous publication discussed the differences in EEG and EMG acquisition for the detection of sleep staging [4]. Respiratory signal differences include *S*_pO_2__ measurement from the forehead, and the utilisation of bioimpedance (BioZ) technology for effort and flow signals. By collecting *S*_pO_2__ from the forehead, the device eliminates the need for a separate component at the fingertip and previous literature suggests that the forehead location may provide a more stable blood flow compared to the fingertip [5–7]. BioZ effort measurement is a noninvasive approach measuring breathing effort by detecting changes in the impedance of the upper chest cavity tissue during breathing. Early validation studies demonstrate a good correlation between bioimpedance measurements and conventional respiratory effort signals, suggesting it could be a viable alternative in both clinical and research settings [8–10].

The aim of this analysis is to validate the accuracy and reliability of the patch-based PSG system against the traditional PSG in assessing respiratory sleep parameters, accounting for system-level differences.

## Materials and methods

### Aims and objectives

This study aimed to validate the scoring of respiratory events recorded by a patch-based sleep monitoring system by comparing them directly to those obtained from a simultaneously worn, traditional PSG device in a sleep laboratory setting. By conducting parallel recordings under controlled conditions, the analysis sought to determine the accuracy, reliability and clinical equivalence of the patch-based system in detecting key respiratory parameters such as apnoeas, hypopnoeas and respiratory effort-related arousals. Particular attention is given to potential discrepancies arising from differences in signal acquisition methods, sensor placement and data resolution between the two systems. The findings will help assess whether the patch-based system can serve as a viable alternative to conventional PSG for respiratory event scoring in both clinical and research contexts.

### Study design

A multicentre study was conducted in Germany at: 1) VAMED Klinik Hagen-Ambrock, 2) Universitätsklinikum Gießen und Marburg, 3) Lungenzentrum Ulm, 4) Evang. Kliniken Essen-Mitte, 5) Universitätsmedizin Essen Ruhrlandklinik, 6) Krankenhaus Bethanien, Solingen, and 7) the American Sleep Clinic, Frankfurt. The study was approved under reference number 2021-2521-evBO by the ethics committee at the chamber of physicians Hessen and local ethics committees as required. Subject recruitment occurred from September 2022 to April 2024. Consecutive participants with suspected sleep disorders who met the inclusion criteria and consented to participate underwent an evaluation in a sleep laboratory using both the patch-based PSG system and a traditional PSG system recorded simultaneously.

#### Inclusion criteria

- 18 years and older
- Referral for a suspected sleep disorder requiring a sleep diagnostic study OR a planned follow-up PSG for an established sleep disorder (without therapy)

#### Exclusion criteria

- Inability to provide informed consent
- History of allergic reactions to adhesives or hydrogels or a family history of adhesive skin allergies
- Severe skin conditions at sites of patch administration
- Implanted cardiac stimulator or diaphragmatic pacer
- An abnormality that made the subject ineligible for inclusion

### Devices

#### Traditional PSG

Traditional PSG studies were conducted using the standard equipment routinely employed at each participating sleep clinic (see supplementary material). A standardised setup included EEG (C3, C4, F3, F4, O1 and O2), left and right EOG (E1-M2 and E2-M1), chin EMG (anterior, left and right positioning), *S*_pO_2__, airflow (nasal pressure), electrocardiography (ECG), breathing efforts (abdomen and thorax), sleeping position, audio and video recording and tibialis EMG on both legs (optional). Minor alterations of some of the electrode positions were made to accommodate the positioning of the patch-based PSG head sensor. The implemented changes had no impact on the integrity or quality of the signal recorded by the traditional PSG system.

#### Patch-based PSG

**FIGURE 1:**
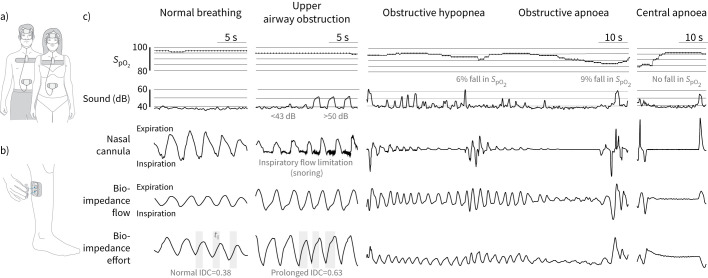
Patch-based PSG application and physiological signals for the diagnosis of sleep disordered breathing. The patch-based type II PSG system consists of four sensors located on the a) head, chest, abdomen and b) leg. The patch-based sensors are comprised of disposable patches and reusable pods that click into the patches for data collection (b). A configuration of the chest-patch signals, oxygen saturation (*S*_pO_2__) (*via* the forehead) and nasal cannula collected using the patch-based device during various sleep disordered breathing events are seen in (c). Under normal breathing there is no airflow obstruction, as highlighted by the normal inspiratory airflow, normal inspiratory duty cycle (IDC) in both the bioimpedance (BioZ) signals and the lack of snoring. Under upper airway obstruction, there are five markers of inspiratory flow limitation: 1) a flattening of the inspiratory airflow, 2) high frequency oscillation in the inspiratory airflow, 3) an early inspiratory peak in airflow and the BioZ flow signal, 4) a prolongation of the inspiratory duty cycle and 5) snoring level exceeding 50 dB. Under both obstructive hypopnea and obstructive apnoea, all the features of upper airway obstruction are present, such as the decline in airflow (incomplete cessation, hypopnoea and complete cessation, apnoea), a fall in oxygen saturation and the presence of breathing effort. Under central apnoea we see a complete cessation in airflow and no breathing effort.

The Onera Sleep Test System (legal manufacturer Onera B.V., Eindhoven, Netherlands), is a self-applied, patch-based type II PSG system designed to record physiological signals during home sleep studies. The EEG (frontal left and right), EOG (left and right), EMG of the masseter muscle (left and right) and *S*_pO_2__ (*via* a reflective pulse oximeter on the forehead) are collected using the head sensor. The *S*_pO_2__ accuracy was independently tested at the University of California San Francisco Hypoxia Lab. The chest sensor collects ECG, respiratory flow (*via* bioimpedance), respiratory effort (*via* bioimpedance), activity (*via* accelerometery), snoring level (*via* sound pressure) and body position (*via* accelerometery). The flow sensor monitors respiratory flow through a nasal cannula. The leg sensor measures EMG of the anterior tibialis muscle. Once the sleep study has been completed, data from each sensor is automatically synchronised and an EDF file is automatically created. Examples of the patch-based PSG setup and respiratory event traces from the EDF file are shown in figure 1.

### Study selection criteria

Study selection criteria were created to ensure that data were representative of a full sleep night. Sleep studies were inspected by a licensed, independent sleep physician using the following criteria:

A) the patch-based PSG study AND the traditional PSG study had ≥6 h of time in bed (TIB); AND
B) ≥4 h of the study recording consisted of sleep (total sleep time (TST)); AND
C) all signals essential for scoring according to American Academy of Sleep Medicine (AASM) criteria were present; AND
D) all signals essential for scoring according to AASM criteria were of scorable quality for at least half of TIB.

The essential signals for the sleep study following 2020 AASM guidelines were: *S*_pO_2__ signal, breathing efforts (thorax and/or abdomen signal), airflow (nasal pressure, optional also thermistor in case of signal deficiencies in nasal pressure), 1 EEG channel (patch-based PSG) or 2 EEG channels (traditional PSG), 1 EOG channel (patch-based PSG) or 2 EOG channels (traditional PSG), EMG chin/masseter, and ECG [11].

If both the patch-based PSG and traditional PSG sleep study met the study selection criteria, the recordings were anonymised, randomised, blinded for device type and sent to three independent, certified sleep professionals: 1) the Johns Hopkins Center for Interdisciplinary Sleep Research and Education in Baltimore, MD, USA; and 2) Sleep Scoring Service LCC in Utah, USA; and 3) a Registered Polysomnographic Technologist (RPSGT) independent sleep scorer in Switzerland. Scorers used RemLogic software (version 1.3, Embla Systems Ltd., Kanata, Canada) to score the studies according to AASM guidelines version 2.6, 2020.

### Sample size calculation

Sample size calculations were performed using the GLIMMPSE online tool [12]. Scorers were treated as a between-subjects factor, while the type of sleep device was modelled as a repeated, within-subjects factor. Prior to initiating the study, conservative assumptions were made regarding expected effect sizes and error variances. It was anticipated that within-subject correlations between the traditional PSG and the patch-based PSG would be stronger than correlations between scorers on either device. Power analyses were conducted across systematic device differences of ±5% in total events per h assuming a normal distribution of AHI with a standard deviation of 20 events per h, a reader-specific standard deviation of 5 events per h, and an alpha level of 0.05 (STATA 17, StataCorp, College Station, TX). The resulting power curves suggested that ∼200–300 paired sleep studies would be required to achieve >0.8 power.

### Analysis

The following variables were calculated: TST, rapid eye movement (REM) time, non-rapid eye movement (NREM) time, AHI, REM AHI, NREM AHI, hypopnoea index (HI), apnoea index (AI), central apnoea index (CAI), obstructive apnoea index (OAI), oxygen desaturation index (ODI), *S*_pO_2__ and arousal index. Hypopnoeas were processed using the AASM manual (v2.6), 3% desaturation or arousal rule, and ODI was calculated by looking at events with desaturation ≥3% [11]. For this analysis, mixed apnoeas and obstructive apnoeas were grouped in the OAI. Arousal differentiation was not conducted as part of the analysis.

The mean±sd for all respiratory event indices was reported for both the traditional PSG and patch-based PSG. The line of best fit was calculated using reduced major axis (RMA) regression and correlation using the concordance correlation coefficient (CCC). RMA and CCC measures account for variation in x (the traditional PSG) and y (test device, patch-based PSG) in their calculations, accounting for the test–retest variability of traditional PSG [13–18]. CCC has been used in previous device validation publications [19–21]. The Altman interpretations of the CCC was used for this analysis [22, 23]. Bland–Altman plots were generated for each metric, displaying the average deviation between device measurements (bias) and 95% confidence interval (limits of agreement).

The interclass correlation coefficient (ICC) was implemented using the pingouin statistical package, version 0.5.5, based on a mean-rating (k=3), absolute, 2-way mixed effects model to assess scorer agreement [24].

Receiver operating characteristic (ROC) curves were used to evaluate the patch-based PSG's ability to distinguish amongst AHI thresholds at 5, 15 and 30. The area under the ROC curve (AUC) was calculated where an AUC of 1 indicates perfect discrimination and an AUC of 0.5 suggests random classification rates [25]. The optimal threshold of the ROC, where sensitivity and specificity are balanced, was calculated using the Youden Index.

All studies were complete. No outlier analysis was performed.

## Results

### Consort diagram

**FIGURE 2:**
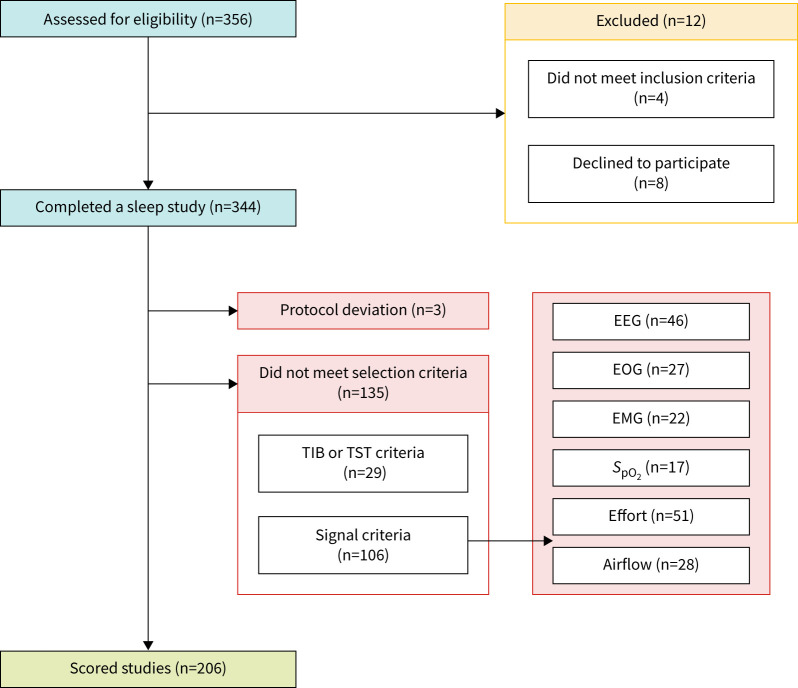
Consort diagram. A total of 206 participants met the study selection criteria and were included in the analysis of the clinical study. Other patients were excluded due to retracting consent, not meeting the inclusion criteria, protocol deviations, sleep time requirements, or signal quality or quantity as outlined by the Study Selection Criteria. TIB: time in bed; TST: total sleep time; EEG: electroencephalography; EOG: electro-oculography; EMG: electromyography; *S*_pO_2__: oxygen saturation.

The study consort diagram is presented in figure 2. Of the 344 subjects who completed an in-lab, side-by-side sleep study, 206 met the predefined criteria for inclusion in the scoring analysis. For further details, refer to the Study Selection Criteria section.

### Demographics

Demographics of the 206 participants can be found in table 1. The demographic characteristics of patients included in the analysis did not differ significantly from those of the excluded patients across all measured variables.

**TABLE 1:** Participant demographics

**Age (years)**	50.9±12.3
* *≥65 years	21 (10.2%)
**Sex (M/F)**	136/70 (66.0%/34.0%)
**BMI kg·m^−2^**	30.2±6.2
**Race**	
Black or African American	6 (2.9%)
White	133 (64.6%)
Not reported/unknown	66 (32.0%)
Other	1 (0.5%)
**Comorbid conditions**	
Cardiac	79 (38.3%)
Pulmonary	30 (14.6%)
Endocrine	58 (28.2%)
Neurological	18 (8.7%)

Demographic information for the 206 subjects included in the analysis. Data are presented as n (%) or mean±sd. M: Male; F: Female; BMI: body mass index.

### Inter-device agreement

Mean sleep and respiratory summary statistics and the CCC between the traditional PSG and patch-based PSG are presented in table 2. Overall, the patch-based PSG demonstrated comparable sleep time and respiratory event rates to the traditional PSG, with strong to very strong agreement (CCC ≥0.7) across nearly all respiratory metrics. Moderate agreement was observed for the HI with a CCC of 0.68. The highest levels of concordance were found in NREM AHI, total AHI, TST and the OAI.

**TABLE 2:** Summary statistics and correlation of respiratory and sleep indices

Variable	Traditional PSG Mean*±*sd	Patch-based PSG Mean*±*sd	Device agreement (CCC)	Traditional PSG interscorer agreement (ICC)	Patch-based PSG interscorer agreement (ICC)
**TST**	358.29±63.40	361.32±66.66	0.87 (0.84, 0.9)	0.96 (0.95, 0.97)	0.93 (0.91, 0.94)
**NREM**	295.32±51.54	295.30±53.93	0.8 (0.75, 0.83)	0.93 (0.91, 0.94)	0.89 (0.86, 0.91)
**REM**	62.97±32.09	66.03±31.97	0.81 (0.76, 0.85)	0.92 (0.9, 0.94)	0.89 (0.86, 0.91)
**AHI**	31.26±23.99	27.75±23.02	0.9 (0.88, 0.92)	0.97 (0.96, 0.98)	0.97 (0.96, 0.97)
**NREM AHI**	30.31±24.92	26.85±23.82	0.91 (0.89, 0.93)	0.97 (0.96, 0.98)	0.96 (0.95, 0.97)
**REM AHI**	35.32±25.03	30.99±23.45	0.8 (0.75, 0.83)	0.95 (0.93, 0.96)	0.93 (0.91, 0.94)
**HI**	19.60±14.91	16.37±13.98	0.68 (0.62, 0.74)	0.86 (0.83, 0.89)	0.76 (0.7, 0.81)
**AI**	11.66±17.60	11.39±18.02	0.89 (0.86, 0.92)	0.95 (0.94, 0.96)	0.95 (0.93, 0.96)
**CAI**	1.63±3.89	2.14±5.63	0.78 (0.69, 0.86)	0.85 (0.81, 0.88)	0.78 (0.72, 0.82)
**OAI**	10.04±16.81	9.25±16.70	0.86 (0.8, 0.9)	0.95 (0.93, 0.96)	0.93 (0.91, 0.94)
**ODI**	25.54±23.17	21.94±20.74	0.79 (0.75, 0.84)	0.96 (0.95, 0.97)	0.98 (0.98, 0.98)
**Arousal index**	25.74±17.70	23.40±16.31	0.72 (0.66, 0.77)	0.53 (0.41, 0.63)	0.52 (0.39, 0.62)

Summary of sleep statistics and respiratory sleep-related events. The CCC was used to account for both the variance in the test device (patch-based PSG) and the traditional PSG device. ICC was used to determine interscorer agreement on each device. Analysis was completed on all scored records (206 patients, triple scored, n=618). PSG: polysomnography; CCC: concordance correlation coefficient; ICC: intraclass correlation coefficient; TST: total sleep time; NREM: non-rapid eye movement; REM: rapid eye movement; AHI: apnoea-hypopnoea index; HI: hypopnoea index; AI: apnoea index; CAI: central apnoea index; OAI: obstructive apnoea index; ODI: oxygen desaturation index.

**FIGURE 3:**
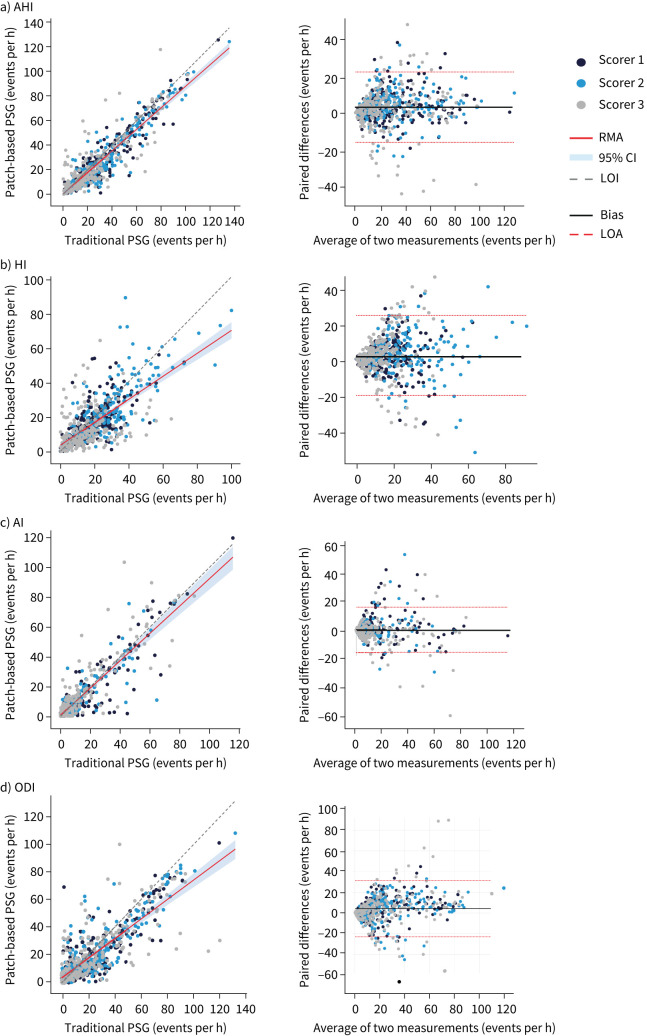
Concordance correlation coefficient (CCC) and Bland–Altman plots for a) apnoea–hypopnoea index (AHI), b) hypopnoea index (HI), c) apnoea index (AI) and d) oxygen desaturation index (ODI). Left-hand panels: CCC for each respiratory index from three scorers comparing the traditional polysomnography (PSG) to the patch-based PSG. Each scorer is represented by a different coloured dot. The line of identity (LOI) is shown as a dotted grey line, and the reduced major axis regression (RMA) is shown as a solid red line. Right-hand panels: Bland–Altman plots for paired respiratory events. The mean offset (bias) between measurements derived from the two devices is shown as a solid black line. The limits of agreement (LOA; the 95% confidence interval of the differences between the two measurement methods) are shown as dashed red lines. Analysis was completed on all scored records (206 patients, triple scored, n=618).

Figure 3 provides an overview of the correlations and Bland–Altman plots for respiratory metrics. In general, the regression analyses show slopes close to the line of identity (LOI) with narrow confidence intervals (CIs). AHI exhibited the smallest CIs and the slope most closely aligned with the LOI, while HI and ODI showed greater deviations from the LOI and wider CIs. Bland–Altman plots revealed low biases (<5), although some metrics, such as AI, demonstrated increased dispersion at higher event rates. For correlation and Bland–Altman plots of OAI and CAI, refer to the supplementary material.

### *S*_pO_2__ comparison

**FIGURE 4:**
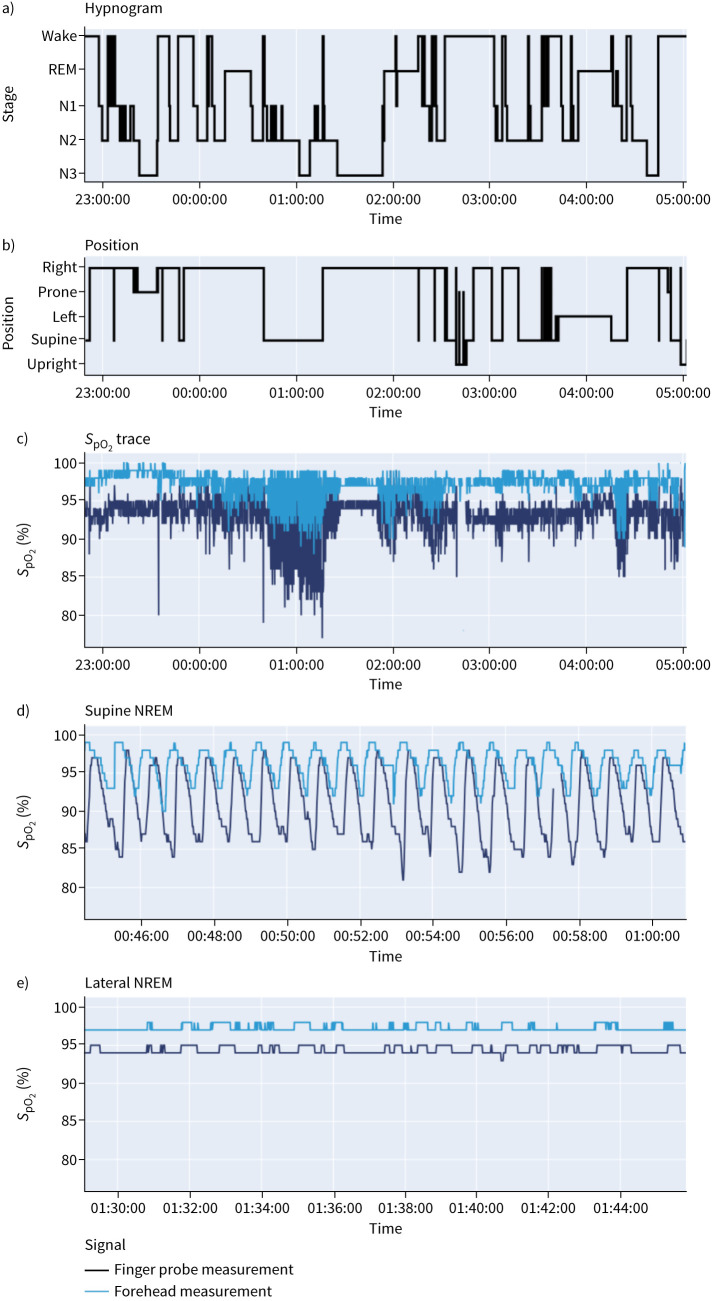
Oxygen saturation (*S*_pO_2__) collection at different measurement sites. A random subject was selected to visualise the finger probe (traditional polysomnography (PSG)) and forehead sensor (patch-based PSG) *S*_pO_2__ measurements simultaneously worn during a sleep night. The patient is a 58-year-old male with a body mass index of 29.7. a) A hypnogram of the sleep night; b) the patient's position measured using the patch-based PSG throughout the whole night; c) the *S*_pO_2__ measurements across the whole night; d) a 15 min segment of *S*_pO_2__ during a supine non-rapid eye movement (NREM) segment; and e) a 15 min segment of *S*_pO_2__ during lateral NREM. The patient has supine and rapid eye movement (REM)-related sleep apnoea as can be seen by the more frequent desaturation events in the supine position. The forehead and finger-probe locations are synchronous, with the forehead location leading the finger probe due to position of the recording site, and the forehead recording higher *S*_pO_2__ measurements with more shallow dips in *S*_pO_2__ during respiratory disturbances.

A comparison of *S*_pO_2__ measurements collected from the forehead (patch-based PSG) and the finger probe (traditional PSG) is presented in table 3. Overall, *S*_pO_2__ values recorded at the forehead were consistently higher across all sleep stages compared to those measured at the finger. For both measurement sites, mean *S*_pO_2__ was lowest during REM sleep. Among patients with significant desaturations, *S*_pO_2__ T90 and *S*_pO_2__ T88 values were comparable between the two sites and showed no statistically significant differences. For a visual representation of *S*_pO_2__ across the different sensor locations, refer to figure 4.

**TABLE 3:** *S*_pO_2__ measurement at different sites

	Forehead	Finger probe	p-value
***S*_pO_2__ overall**	94.7±2.1	93.0±2.2	<0.001^#^
***S*_pO_2__ wake**	95.5±1.9	93.8±2.0	<0.001^#^
***S*_pO_2__ NREM**	94.6±2.1	92.9±2.1	<0.001^#^
***S*_pO_2__ REM**	93.6±3.0	92.6±3.1	<0.001^#^
***S*_pO_2__ T90 (min)**	47.6±103.3	58.8±92.7	0.245
***S*_pO_2__ T88 (min)**	26.2±74.4	25.9±57.2	0.965
**ODI overall**	22.04±20.78	26.27±23.95	0.001^#^
**ODI NREM**	20.36±21.33	24.70±24.62	0.001^#^
**ODI REM**	28.93±22.88	33.47±26.82	0.002^#^

*S*_pO_2__ was compared at the two measurement sites, forehead (patch-based PSG) and finger probe (traditional PSG). Results are reported as mean±sd of *S*_pO_2__ values except in the case of *S*_pO_2__ T90 and T88 in which the mean amount of time (in minutes) below an *S*_pO_2__ value of 90% and 88% was reported. Analysis was completed on n=206 subjects comparing the patch-based and traditional PSG records. *S*_pO_2__: oxygen saturation; ODI: oxygen desaturation index; NREM: non-rapid eye movement; REM: rapid eye movement; PSG: polysomnography. ^#^: indicates a statistically significant difference between measurement sites computed using a t-test with cut-off value at p=0.05.

### Inter-scorer agreement

All variables (except the arousal index) showed high agreement (≥0.70) on both devices. The arousal index showed low agreement (0.53 traditional PSG, 0.52 patch-based PSG) for both devices. On average, agreement among scorers was higher on the traditional PSG than on the patch-based PSG, but most results were comparable between devices (<0.05 difference). The patch-based PSG had higher interscorer agreement for OAI, and equal ICC for AHI and AI compared to the traditional PSG.

### Receiver operating characteristic

**FIGURE 5:**
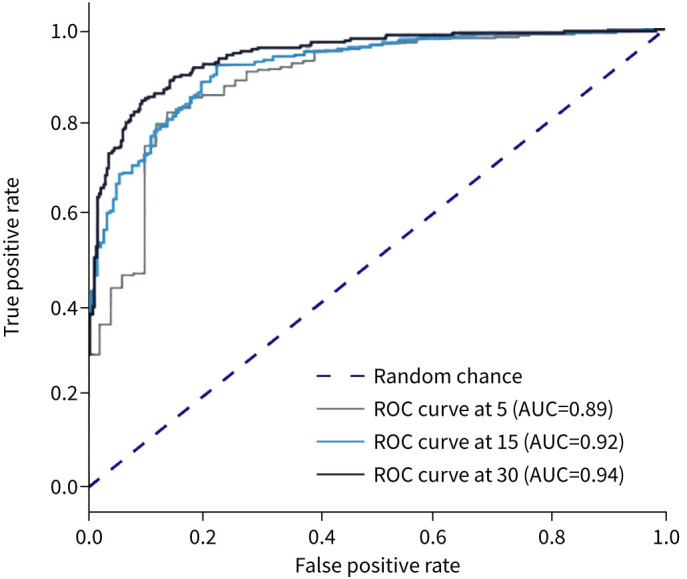
Receiver operating characteristic (ROC) curves illustrating the patch-based device diagnostic performance for detecting obstructive sleep apnoea at apnoea–hypopnoea index (AHI) thresholds of 5, 15 and 30 events per h. The areas under the curve (AUCs) are 0.89, 0.92 and 0.94, respectively, indicating high discriminative ability across mild, moderate and severe disease cut-offs. The diagonal dashed line represents random chance.

The ROC curves are visualised for the three AHI severity thresholds (≥5, ≥15, ≥30) in figure 5. There were 564 scored traditional PSG studies with AHI ≥5, 431 with AHI ≥15 and 249 with AHI ≥30. All three curves (thresholds) approach the top left corner of the graph (perfect prediction) and showed excellent discrimination between classes (AUC ≥0.8).

### Protocol deviations and adverse events

Adverse events were reported in 23 instances (6.2%), none of which were serious cases. Of these, 20 (5.9%) were attributed to the patch-based PSG, predominantly mild skin reactions (reddening, irritation, allergic response), with one prolonged allergic reaction lasting >1 week. The reported events were consistent with the expected categories. Two events were unrelated (COVID-19 and common cold). All events resolved with follow-up.

## Discussion

The results of our study demonstrate that despite design differences, the patch-based PSG system provides diagnostic utility in distinguishing OSA severity at all AHI thresholds (AUC ≥0.8) and a strong–very strong ability to distinguish between different respiratory metrics (AHI, AI, OAI, CAI, ODI, arousal index CCC ≥0.7) when compared to traditional PSG.

Respiratory signal design changes in the patch-based PSG for increased usability was a single BioZ patch-based effort measurement and *S*_pO_2__ collection *via* the forehead. The results of this clinical study indicate that BioZ effort, as part of a respiratory measurement signal set including a nasal cannula, *S*_pO_2__, and snoring sound pressure, can be used to reliably distinguish respiratory events during sleep as seen by the strong OAI and CAI correlations (≥0.7 CCC).

The patch-based PSG uses a forehead *S*_pO_2__ sensor. The forehead site has rich vascularisation ensuring stable blood flow and reducing measurement inaccuracies due to poor perfusion [6, 7]. Despite fewer desaturation events recorded by the patch-based PSG—likely due to physiological differences in sensor placement—the forehead-derived ODI showed strong correlation and HI showed moderate correlation with traditional finger-probe measurements. These differences in measurement location suggest that the assessment of hypoxic burden is influenced by the site of oximeter placement, which may have implications for research on hypoxic burden and its relationship to cardiovascular outcomes [26]. Regardless, forehead *S*_pO_2__ monitoring may be especially suitable for sleep apnoea patients with significant comorbidities that may compromise blood flow in the extremities [5, 6].

### Study limitations and implications

This clinical study has several limitations. Although the study population was representative of the participating clinical sites, it primarily included individuals with mild obesity and lacked reported racial diversity. Given known inaccuracies in *S*_pO_2__ measurements among individuals with darker skin tones, future research is needed to ensure reliable oxygen saturation readings across diverse patient populations [27].

Additionally, the study focused on validating the patch-based PSG in a general sleep-disordered cohort. Further investigations are warranted to evaluate its performance in specific clinical subgroups. Furthermore, the patch-based PSG was manually scored in this study; whether automatic analysis of the recordings can yield comparable results requires further investigation.

The study experienced a high attrition rate, which was partly intentional due to stringent selection criteria designed to ensure robust signal comparisons. As a result, some patients with potentially clinically useful data were excluded. Another source of attrition stemmed from the design of the patch-based PSG device. Unlike traditional in-lab PSG, technicians could not monitor or correct sensor detachments during the study, as the patch-based system is intended for unattended home use. Similar attrition rates and selection methods have been reported in other device validation studies [28]. To mitigate this, patient recruitment continued until the required sample size was achieved based on power calculations. Therefore, the attrition rate did not compromise the validity of the findings and is not reflective of the device's performance in its intended home-use setting.

It is important to note that the performance of the patch-based PSG may differ when self-applied at home. This study did not investigate patient-led application, and real-world use may introduce additional challenges. Social and environmental factors—such as co-sleeping, alcohol consumption and smoking—can influence data quality and interpretation. Ongoing studies are evaluating the device's performance in home environments with patient self-application.

The findings of this study carry important implications for clinical practice. In-lab PSG currently falls short of meeting diagnostic demand, which is expected to increase significantly over the next decade [29–32]. Limitations in the availability of PSG beds, devices and trained personnel hinder the ability to scale in-lab testing. Alternative solutions, such as home-based PSG with patient self-application, must be considered to address this growing need. Historically, most PSG systems require technician involvement for setup and removal, even in home settings [31, 33]. However, newer technologies for self-applied home PSG are available and have been validated [34].

Beyond addressing capacity constraints, home-based PSG offers several advantages. Testing in a familiar environment may better reflect natural sleep patterns and improve accessibility for patients in rural or underserved areas. Decentralised testing also has the potential to reduce diagnostic wait times, enable earlier detection of sleep disorders and accelerate treatment initiation. Collectively, these benefits suggest that home-based PSG may serve as a valuable complement to traditional in-lab diagnostics.

### Conclusion

The patch-based PSG system demonstrated strong–very strong agreement with traditional PSG systems in measuring sleep-disordered breathing, accurately distinguishing between different types of sleep disordered breathing events and in identifying sleep apnoea at different AHI thresholds. Using the patch-based PSG to perform self-applied, at-home, unsupervised PSG studies may support the growing demand for sleep studies.

## Data Availability

Data from this clinical study are sensitive and will not be made publicly available.

## Supplementary material

**Please note:** supplementary material is not edited by the Editorial Office, and is uploaded as it has been supplied by the author. Supplementary figure 00857-2025.SUPPLEMENT Supplementary table 00857-2025.SUPPLEMENT
