# Supplementary material for: Clinical validation of respiratory outcomes for a patch-based polysomnography system
Source: ERJ Open Res. 2026 Jun 8;12(3):00857-2025. doi: 10.1183/23120541.00857-2025 (PMC13244203; doi:10.1183/23120541.00857-2025)
Supplement: Supplementary file 2 [file 00857-2025.SUPPLEMENT.pdf]

Clinic	Count (%)	PSG System(s)
American Sleep Clinic, Frankfurt	56 (16.3%)	Embla S4500 Natus Medical Incorporated
Evang. Kliniken Essen-Mitte	33 (9.6%)	Loewenstein Medical MiniScreen
VAMED Klinik Hagen-Ambrock	71 (20.6 %)	Alice 5 Philips Respironics
Universitätsmedizin Essen Ruhrlandklinik	35 (10.2%)	Nox A1
Lungenzentrum Ulm	49 (14.2%)	Nox A1
Krankenhaus Bethanien	27 (7.8%)	Alice 6 LDe, SONATA Löwenstein Medical
Universitätsklinikum Marburg	73 (21.2%)	Sleep Doc Porti
